# P-1549. Intravenous-line Related Complications in Patients Treated with Short-term Intravenous Antibiotics for Complicated Urinary Tract Infection and Other Infections: A Systematic Literature Review

**DOI:** 10.1093/ofid/ofae631.1716

**Published:** 2025-01-29

**Authors:** Fanny S Mitrani-Gold, Myriam Drysdale, Saifuddin Kharawala, Pooja Malhotra, Neeti Chana, Jeffrey J Ellis, Alanna Farrell-Foster, Emily Lloyd

**Affiliations:** GlaxoSmithKline plc., Collegeville, Pennsylvania; GSK, Brentford, Middlesex, England, United Kingdom; Bridge Medical Consulting Limited, London, England, United Kingdom; Bridge Medical Consulting Limited, London, England, United Kingdom; Bridge Medical Consulting Limited, London, England, United Kingdom; GSK, Brentford, Middlesex, England, United Kingdom; GSK, Brentford, Middlesex, England, United Kingdom

## Abstract

**Background:**

Complicated urinary tract infections (cUTI) often involve multidrug-resistant uropathogens requiring treatment with intravenous (IV) antibiotics (ABX); risk of IV-line complications increases with longer treatment duration. We studied the occurrence and impact of IV-line complications in patients with cUTI and other infections treated with IV ABX for < 30 days.

**Figure:**
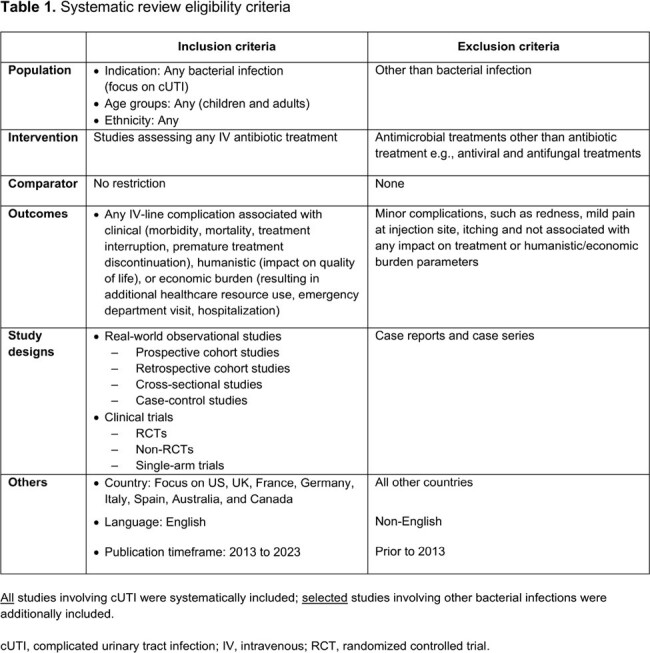

**Methods:**

A systematic literature search for cUTI was conducted in MEDLINE and Embase, supplemented by conference, bibliography, and keyword-based searches (2013–2023). Additional searches on other bacterial infections requiring short term IV ABX were performed due to limited cUTI data. All cUTI primary publications/studies and studies prioritized for other infections were included.

**Figure:**
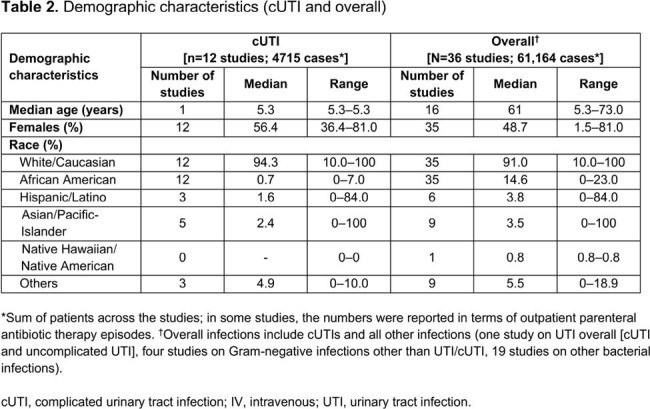

**Results:**

We identified 130 publications, with 36 eligible primary studies included for evidence synthesis (**Table 1**). Most publications (n=30) involved patients from the US and/or Europe. Mean/median age range was 5.3–73.6 years (**Table 2**). Most (median 91.0%) patients were White/Caucasian. At least one IV-line complication was reported in the overall population (median 6.7% [range 0–38.0%]) and in patients with cUTI (median 1.7% [range 0–3.3%]; **Table 3**). Overall, more serious IV-line complications (median [range]) included bloodstream infections (1.2% [0–3.0%]) and thrombosis/thromboembolism (1.1% [0–3.5%]). For any IV-line complication, the median incidence rate was 3.7 per 1000 catheter days (95% CI, 0.5–15.6; **Table 4**); the most frequent IV complications were bloodstream infections and displacement/dislodgement (0.6 per 1000 catheter days, each). IV complication rates in patients with cUTI were generally comparable with those in the overall sample and were lower in patients who transitioned from IV to oral ABX, vs those who continued to receive IV ABX. IV complications resulted in increased clinical and economic burden in the form of IV replacement, premature IV removal, emergency department visit, extended hospital stay, readmission, intensive care unit admission, and increased mortality.

**Figure:**
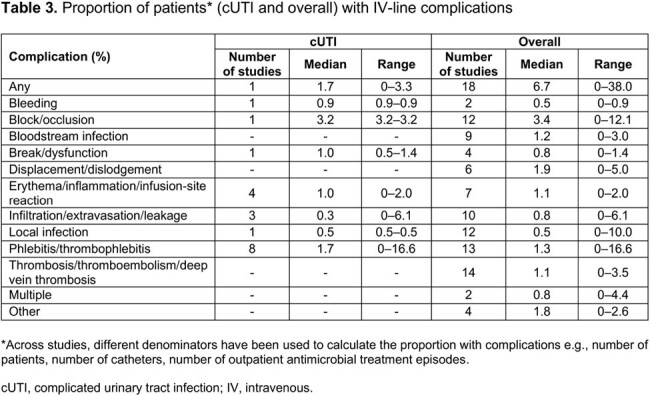

**Conclusion:**

IV-line complications in patients receiving short term IV ABX can result in an increased healthcare burden; complications are less frequent in patients who transition from IV to oral ABX.

**Funding:** GSK study 221810

**Figure:**
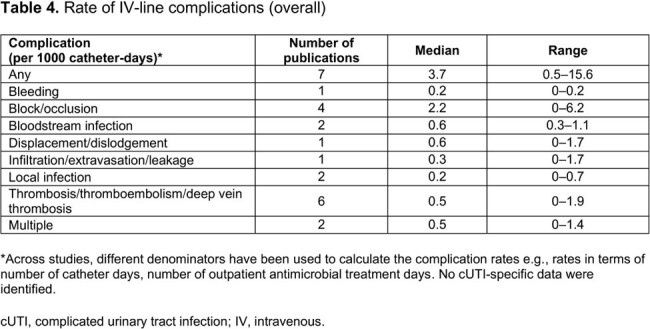

**Disclosures:**

**Fanny S. Mitrani-Gold, MPH**, GSK: Employee|GSK: Stocks/Bonds (Public Company) **Myriam Drysdale, PhD**, GSK: Employee|GSK: Stocks/Bonds (Public Company) **Saifuddin Kharawala, DPM**, Bridge Medical Consulting Ltd.: Employee of Bridge Medical Consulting Ltd., who received funding from GSK to complete this study|GSK India: Previous employee **Pooja Malhotra, MPharm**, Bridge Medical Consulting Ltd.: Employee of Bridge Medical Consulting Ltd., who received funding from GSK to complete this study **Neeti Chana, MPharm**, Bridge Medical Consulting Ltd.: Employee of Bridge Medical Consulting Ltd., who received funding from GSK to complete this study **Jeffrey J. Ellis, PharmD, MS**, GSK: Employee|GSK: Stocks/Bonds (Public Company) **Alanna Farrell-Foster**, GSK: Employee|GSK: Stocks/Bonds (Public Company) **Emily Lloyd, MSc**, GSK: Employee|GSK: Stocks/Bonds (Public Company)

